# Planar Tetracoordinate Silicon in Si_3_Cu_3_^−^ Cluster

**DOI:** 10.1002/anie.202415789

**Published:** 2024-11-07

**Authors:** Xiao‐han Yin, Hong‐lin Zeng, Xin‐bo Liu, Xi‐Ling Xu, Hong‐Guang Xu, Gabriel Merino, Wei‐jun Zheng, Zhong‐hua Cui

**Affiliations:** ^1^ Institute of Atomic and Molecular Physics Jilin University Changchun 130023 China; ^2^ Beijing National Laboratory for Molecular Sciences State Key Laboratory of Molecular Reaction Dynamics Institute of Chemistry Chinese Academy of Sciences Beijing China; ^3^ Departamento de Física Aplicada Centro de Investigación y de Estudios Avanzados Unidad Mérida. Km 6 Antigua Carretera a Progreso. Apdo. Postal 73, Cordemex 97310 Mérida Yuc. México; ^4^ Key Laboratory of Physics and Technology for Advanced Batteries (Ministry of Education) Jilin University Changchun 130023 China

**Keywords:** planar tetracoordinate silicon, photoelectron spectroscopy, global minimum, chemical bonding

## Abstract

Photoelectron spectroscopy and theoretical calculations have identified the global minimum structure of the 16‐valence electron Si_3_Cu_3_^−^ cluster, which features a planar tetracoordinate silicon (ptSi) in a rhombic arrangement. The Si_3_ and Cu_3_ triangles are interconnected by a Si_2_/Cu_2_ edge, forming an ordered chain‐like structure. Besides the conventional 2c–2e σ‐bond connecting Si_3_ and Cu_3_, the stability of this cluster is reinforced by a delocalized 3c–2e σ‐bond in Cu_3_ and a π‐bond in Si_3_. Our study provides experimental confirmation of a planar hypercoordinate heavier Group 14 element, opening possibilities for exploring similar structures in two‐dimensional materials.

## Graphical abstract

Photoelectron spectroscopy and theoretical calculations identified the global minimum structure of the 16‐valence electron Si_3_Cu_3_^−^ cluster, characterized by a planar tetracoordinate silicon atom within a rhombic framework of alternating Si_3_ and Cu_3_ units. The stability is reinforced by a delocalized 3‐center 2‐electron σ‐bond in the Cu_3_ unit and a π‐bond within the Si_3_ framework. This study provides experimental confirmation of a planar hypercoordinate Group 14 element.

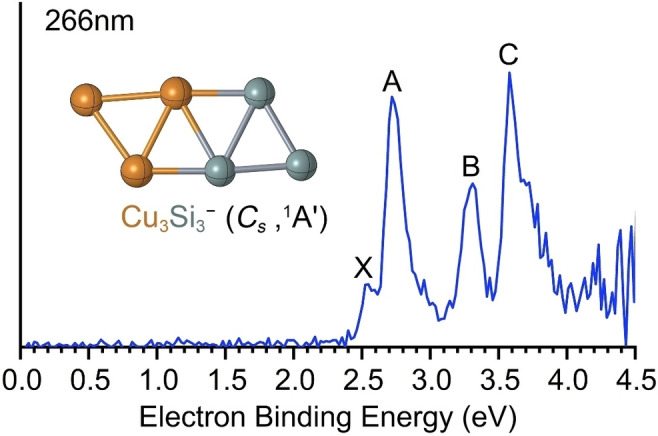

The experimental realization of planar tetracoordinate carbon (ptC)[^1^, ^2^, ^3^] challenged the belief that carbon could only form four single bonds with a tetrahedral geometry.^[4]^ Initially proposed by Hoffmann, Alder, and Wilcox, this concept marked a shift in our understanding of unusual carbon structures.^[5]^ Over five decades, the perception of ptC structures evolved from being considered transition states by Monkhorst,^[6]^ to high‐lying isomers,^[7]^ and eventually as low‐lying forms[^8^, ^9^] or even lowest‐energy isomers,[^10^, ^11^, ^12^, ^13^, ^14^, ^15^] making them strong candidates for experimental validation.

Gas‐phase photoelectron spectroscopy (PES) has detected ptC structures in clusters like CAl_4_^−^,^[16]^ CAl_4_^2−^,^[17]^ CAl_3_Si^−^,^[18]^ CAl_3_Ge^−^,^[18]^ CAl_11_^−^,^[19]^ C_5_Al_5_^−^,^[20]^ and CAl_4_H^−^.^[21]^ The success of ptC research led to exploring heavier Group 14 analogues.[^22^, ^23^, ^24^, ^25^, ^26^] Early attempts to incorporate planar tetracoordinate silicon (ptSi) into organic ligands were initially unsuccessful.[^27^, ^28^] In 1979, the group of Schleyer predicted a ptSi structure in orthosilicic acid esters,^[29]^ but Hönle et al. showed that the orthosilicate of catechol forms a one‐dimensional polymer with tetrahedrally bound silicon in the crystal structure.^[30]^ Recent studies confirmed that while monomeric bis(catecholato)silane adopts a tetrahedral geometry in the gas phase, it becomes metastable and oligomerizes in the condensed phase depending on the catechol, temperature, and concentration.^[31]^

In 2000, Wang and co‐workers provided the first experimental evidence of ptSi in the SiAl_4_^−^ cluster using PES experiments and theoretical computations (see **A** in Scheme 1).^[32]^ This work prompted theoreticians to design ptSi and higher‐coordinate silicon species. Later studies explored planar geometries in boron rings^[33]^ and polygonal hydro‐transition‐metal complexes,[^34^, ^35^, ^36^] identifying local minima for ptSi and other higher‐coordinated silicon centers. In 2004, Li et al. proposed a structural model with planar tetra‐, penta‐, hexa‐, hepta‐, and octacoordinate silicon in the *C*_2v_ B_n_E_2_Si series (E=CH, BH, or Si; *n*=2–5), and (B_n_E_m_Si)_2_H_2_ molecules (S‐shaped or cyclic).[^37^, ^38^] Recent predictions include globally stable planar pentacoordinate silicon (ppSi) in XMg_4_Y^−^ (X=Si, Ge; Y=In, Tl)^[39]^ and planar hexacoordinate silicon (phSi) in SiSb_3_M_3_^+^ (M=Ca, Sr, Ba) (see **D** and **E** in Scheme 1).^[40]^

**Scheme 1:**
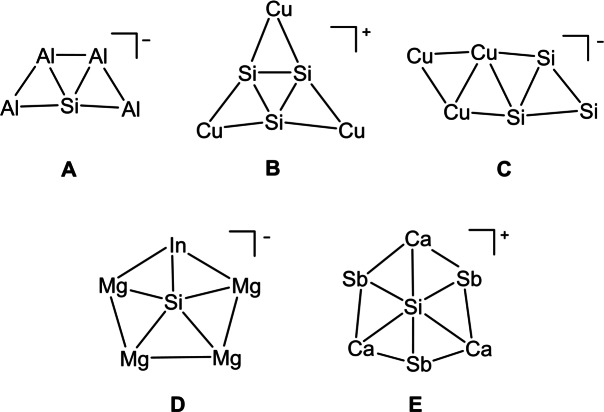
Lowest‐energy structures of planar hypercoordinate silicon clusters.

Several low‐energy ptSi structures have been predicted, including X_3_M_3_^+^ (M=Cu, Li) as shown in **B** in Scheme 1,[^41^, ^42^] XIn_4_^2−^,^[43]^ XB_2_Be_2_,^[44]^ Ca_3_SiAl^−^,^[45]^ Mg_4_Si^2−^,^[45]^ and C_2_Si_2_X^q^ (q=+1, 0, −1; X=heavier Group 14 elements).^[46]^ However, experimental verification of these structures has remained challenging. Recently, the synthesis of ptSi species embedded in organic ligands has been reported.[^47^, ^48^, ^49^, ^50^]

In our search for suitable candidates for gas‐phase ptSi structures, we focused on simple compositions to facilitate detection. We investigated doping Si_3_ with Cu atoms, which have a similar atomic radius to Si. This approach led to the Cu_n_Si_3_^−^ (*n*=2, 3) anions. Potential energy surface analyses revealed that both Cu_2_Si_3_^−^ and Si_3_Cu_3_^−^ can adopt a ptSi configuration. Interestingly, the ptSi form is the second lowest‐energy structure for Si_3_Cu_2_^−^ and the global minimum for Si_3_Cu_3_^−^. Photoelectron spectroscopy confirmed the ptSi structure in Si_3_Cu_3_^−^.

The Si_3_Cu_3_^−^ cluster exhibits a *C_s_* rhombic arrangement, with triangular Si_3_ and Cu_3_ units connected by their Si_2_ and Cu_2_ edges, forming an ordered chain‐like structure (see **C** in Scheme 1). The stability of the ptSi arises from a combination of bonding interactions, including a two‐center two‐electron (2c–2e) Cu−Si σ‐bond between the Si_3_ and Cu_3_ units, a 3c–2e σ‐bond within the Cu_3_ unit, and a π‐bond within the Si_3_ motif. These interactions are key to stabilizing the ptSi form of Si_3_Cu_3_^−^.

The potential energy surfaces of the Cu_n_Si_3_^−^ (*n*=2, 3) anions were explored using the GASA (Genetic Algorithm and Structure‐driven Approaches for atomic clusters) program.^[51]^ The initial geometries for the doublet and quartet states of Cu_2_Si_3_^−^ and the singlet and triplet states of Si_3_Cu_3_^−^ were optimized at the PBE0^[52]^‐D3^[53]^/def2‐SVP^[54]^ level. These geometries were then refined at the PBE0‐D3/def2‐TZVP^[54]^ level. Single‐point energy calculations were performed at the CCSD(T,full)^[55]^/def2‐TZVP level for the low‐lying energy minima identified at the PBE0‐D3/def2‐TZVPP^[54]^ level to obtain more accurate relative energies.

Figure S1 depicts the low‐lying isomers of Cu_2_Si_3_^−^. The lowest‐energy isomer is a three‐dimensional *C_s_* structure in the doublet state, with a relative energy of 1.9 kcal/mol lower than the second lowest‐energy isomer, which adopts a ptSi configuration at the CCSD(T,full)/def2‐TZVP level. All other doublet and quartet isomers are at least 6.9 kcal/mol higher in energy than the ptSi structure.

The low‐lying isomers of Si_3_Cu_3_^−^ are shown in Figure 1, where the ptSi form is the global minimum with the lowest vibrational frequency of 40 cm^−1^. The nearest 3D isomer is higher in energy than the ptSi structure by 1.4 kcal/mol at the CCSD(T)/def2‐TZVP level and by 1.7 kcal/mol at the CCSD(T)/aug‐cc‐pVQZ level. All other 3D isomers are at least 4.6 kcal/mol less stable than the ptSi structure. The lowest‐energy triplet is 14.3 kcal/mol higher in energy than the ptSi form. All structures exhibit reliable *T*_1_ diagnostic values (<0.03) for the converged CCSD wavefunction, indicating minimal multireference character.

**Figure 1:**
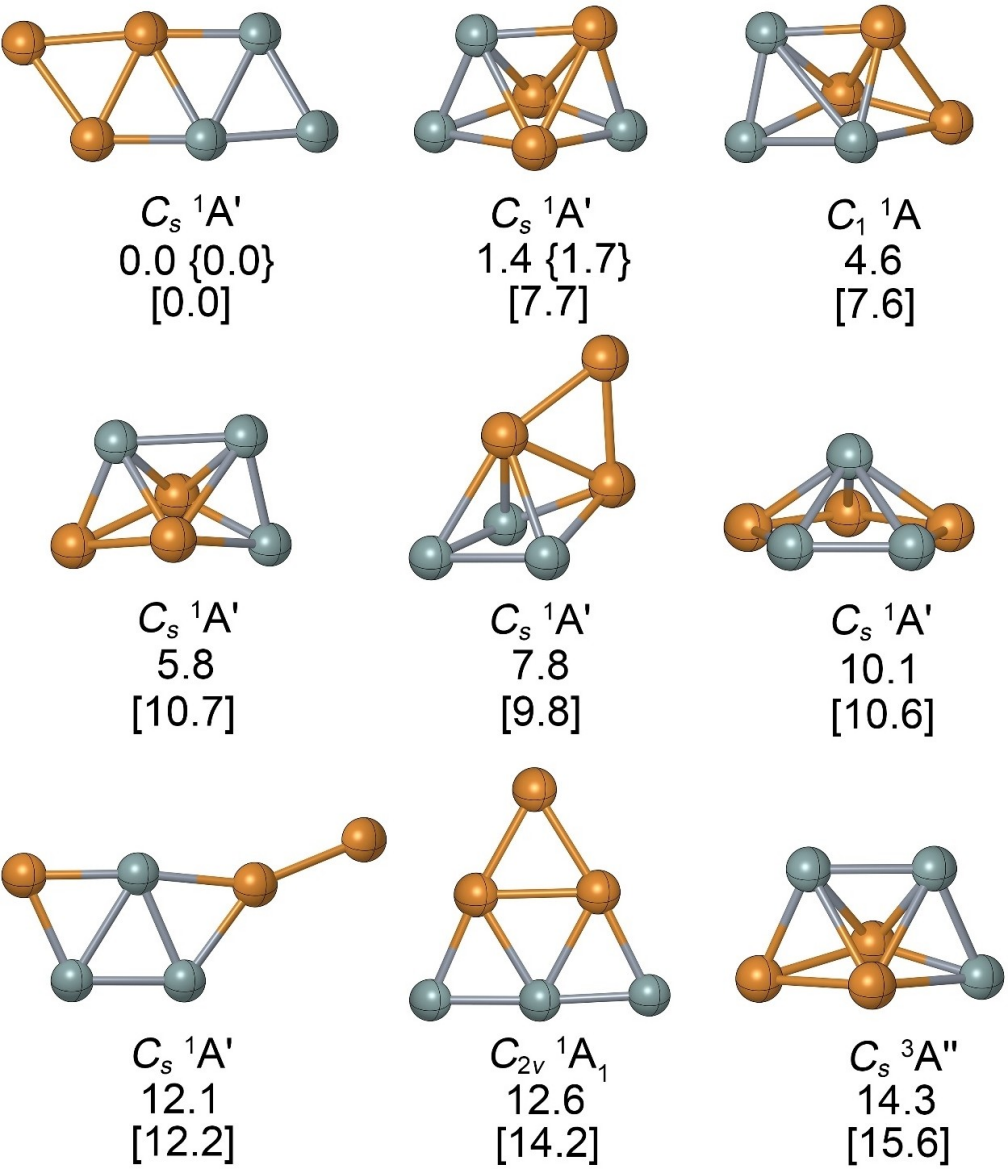
Structures and relative energies (in kcal/mol) of the low‐lying energy isomers of Si_3_Cu_3_^−^ calculated at the CCSD(T,full)/def2‐TZVP//PBE0‐D3/def2‐TZVP level. The energy values from PBE0‐D3/def2‐TZVP and CCSD(T)/aug‐cc‐pVQZ single‐point calculations are provided in square brackets and curly brace, respectively.

Figure 2 summarizes the geometrical parameters of Si_3_Cu_3_^−^ calculated at the PBE0‐D3 level. Two different Si−Cu bond lengths of 2.29 and 2.49 Å are obtained, comparable to the Si−Cu single bond length of 2.28 Å based on self‐consistent covalent radii by Pyykkö.^[56]^ Cu−Cu and Si−Si bond lengths also fall within the range of single bond lengths (2.23 to 2.48 Å). In Si_3_Cu_3_^−^, the Si atoms hold negative charges, with natural population analysis (NPA) charges of −0.16, −0.45, and −0.70 |e| (see Figure S2). In contrast, the Cu atoms bonded to Si carry positive charges of approximately +0.35 |e|, while the dicoordinate Cu atom has a charge of −0.39 |e|, indicating a charge transfer from Cu_3_ to the Si_3_ motif.

**Figure 2:**
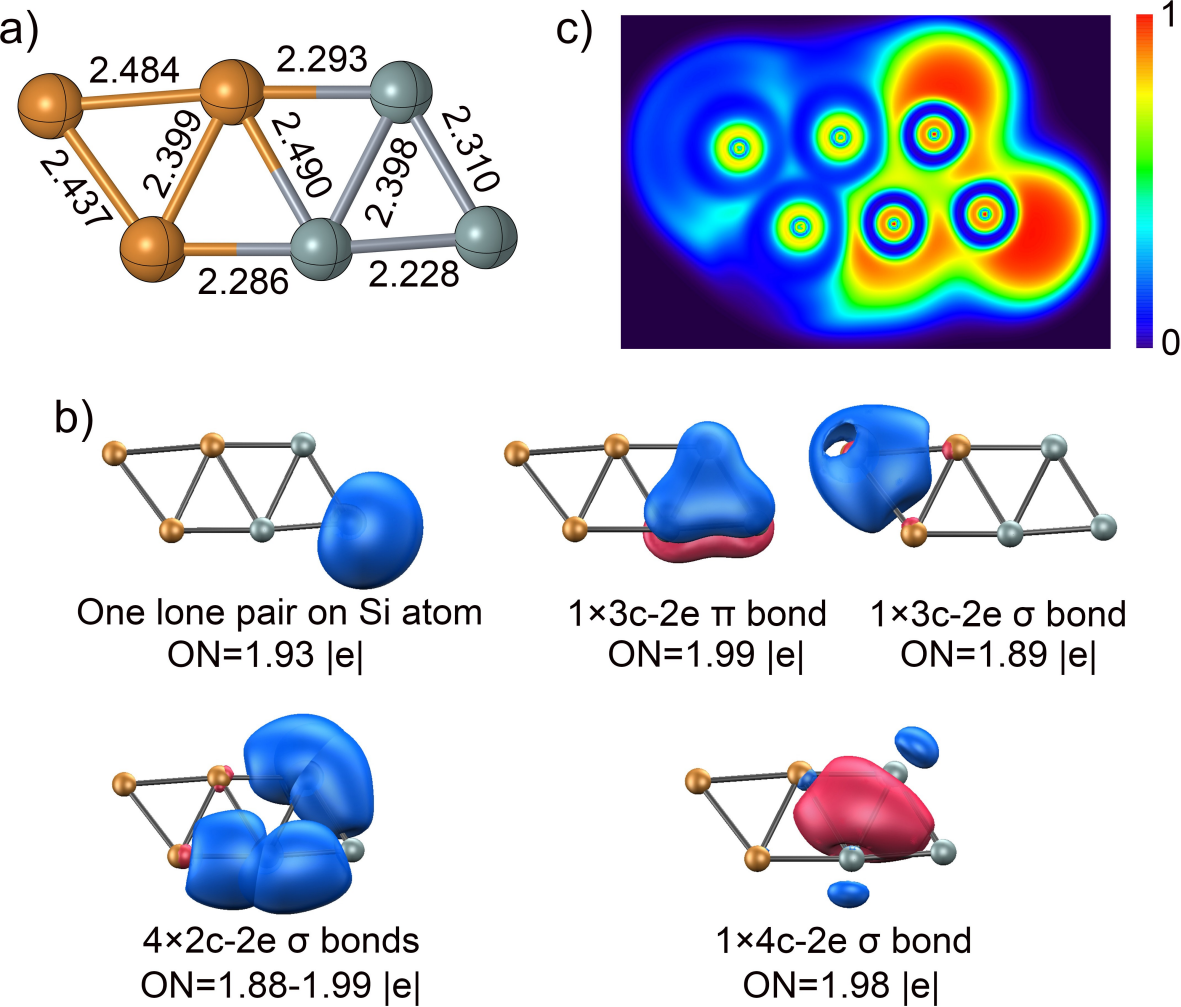
a) Bond lengths (in Å), b) Adaptive Natural Density Partitioning (AdNDP) analysis, and c) Electron Localization Function (ELF) of Si_3_Cu_3_^−^, calculated at the PBE0‐D3/def2‐TZVP level.

To gain a deeper insight into the bonding nature and stability of Si_3_Cu_3_^−^, we performed an adaptive natural density partitioning (AdNDP) analysis (Figure 2b).^[57]^ The top row of Figure 2b shows the lone pair localized at the apical Si atom with an occupation number (ON) of 1.93|e|. The shorter peripheral Si−Si and Si−Cu bonds are attributed to strong 2c–2e σ‐bonds with high (ON=1.88–1.99|e|). Additionally, the structure features a 3c–2e π‐bond within the triangular Si_3_ unit, complemented by 3c–2e σ‐bonds within the triangular Cu_3_. The ON (1.98|e|) for the 4c–2e σ bonds highlights electron delocalization across the cluster. So, this π+σ delocalization contributes to the stability of Si_3_Cu_3_^−^. The AdNDP analysis is consistent with the electron localization function (ELF),[^58^, ^59^] as shown in Figure 2c. The ELF indicates strong electron localization at the Cu_3_ motif and a lone pair at the apical dicoordinate Si atom, with additional electron accumulation at the upper right tricoordinate Si atom, contributing to the localized Si−Si and Si−Cu bonds rather than a fully localized lone pair on the Si atom. The ELF map in Figure S3, taken 1 Å above the molecular plane, further shows the π‐delocalization within the Si_3_ motif and the presence of a lone pair on the dicoordinate Si atom.

In SiAl_4_^−^,^[8]^ a 17‐valence electron system, the cluster is stabilized by π‐delocalization and σ‐donating and π‐accepting substituents. The size of the Al atoms creates a small cavity to accommodate second‐ or third‐row central atoms, leading SiAl_4_^−^ to adopt a planar trapezoidal structure instead of a square one.^[32]^ Increasing the number of hypercoordination or adding ligands not directly bonded to the planar hypercoordinate center may require stabilization beyond the 17‐valence electrons. In such cases, π‐delocalization is not always uniformly distributed across the molecular plane, and σ‐delocalization may also contribute to stabilization. In Si_3_Cu_3_^−^, π‐delocalization involving the ptSi atoms is mainly confined to the Si_3_ units. Note that the ptSi atom is surrounded by four bonds (or eight electrons): a 4c–2e σ‐bond involving the ptSi atom, a 3c–2e σ‐bond within the Cu_3_ triangle, and two Si−Si σ‐bonds. These features result in the unique ptSi cluster, which contains 16‐valence electrons.

Figure 3 displays the photoelectron spectra of Si_3_Cu_3_^−^ recorded at two wavelengths (355 and 266 nm). Briefly, the Si_3_Cu_3_^−^ cluster was generated in a laser vaporization source using laser ablation of a rotating and translating disk target (13 mm diameter, Cu : Si mole ratio of 5:1) with second harmonic (532 nm) light pulses from a Nd : YAG laser. The observed spectral bands are labeled (X, A, B, C) in the Figure, and the corresponding vertical detachment energies (VDEs) are listed in Table 1, which compares the experimental VDEs with theoretical results for the lowest‐energy ptSi isomer and its closest‐energy isomer. Band X corresponds to the first VDE (VDE_1_), representing electron detachment from the ground state of the anion to form the neutral species, and is relatively weak with a VDE of 2.57 eV at 355 nm. Bands A, B, and C represent detachment transitions to various excited states. Band A features an intense and sharp peak with a VDE of 2.74 eV, while an additional intense peak is observed near the detachment threshold at 355 nm, with a VDE of 3.32 eV. The spectrum obtained at 266 nm reveals a well‐defined band C with a VDE of 3.58 eV.

**Figure 3:**
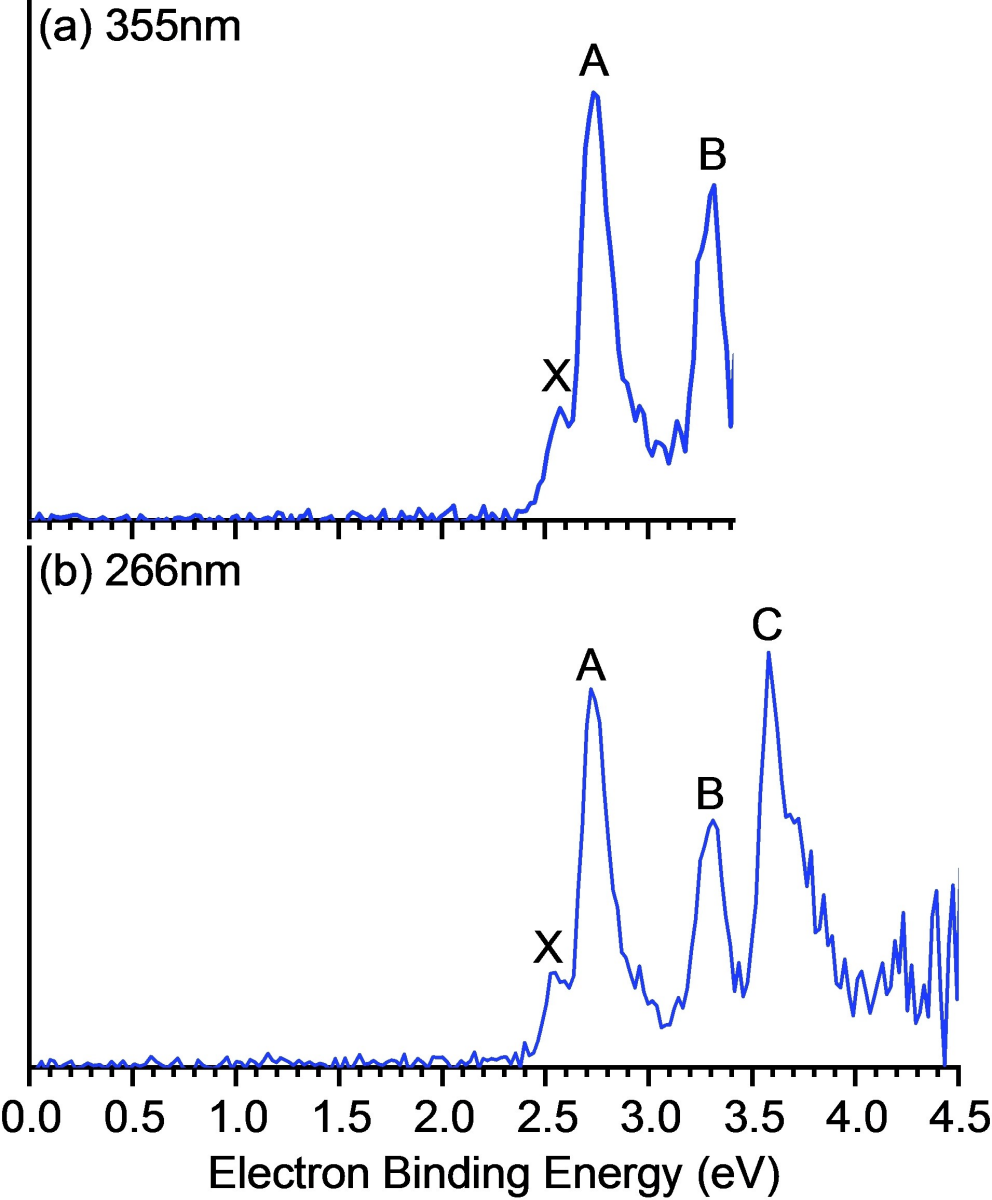
Photoelectron spectra of Si_3_Cu_3_^−^ cluster measured with 266 and 355 nm photons.

**Table 1:** Theoretical VDEs of the ptSi Si_3_Cu_3_^−^ isomer using the EE‐EOM‐CCSD(t)(a)*/def2‐TZVP level compared to the experimental VDEs.

Peak	VDE (eV) (Expt.)	Final state	Electronic configuration	VDE (eV) (Theor.) EOM‐CCSD(t)(a)*
X	2.57	^2^A′	13a′^2^7a“^2^14a′^2^15a′^2^ **16 a’** ^1^	2.47
A	2.74	^2^A′	13a′^2^7a“^2^14a′^**2**^ **15 a’** ^1^16a′^2^	2.98
B	3.32	^2^A′	13a′^2^7a“^2^ **14 a’** ^1^15a′^**2**^16a′^2^	3.36
C	3.58	^2^A′′	13a′^2^ **7a“** ^1^14a′^2^15a′^2^16a′^2^	3.52

To compute the higher detachment channels in the photoelectron spectra, several theoretical approaches were employed, including time‐dependent density functional theory (TD‐DFT),^[60]^ excited states equation‐of‐motion coupled‐cluster single and double (EE‐EOM‐CCSD(t)(a)*)^[61]^ with the continuum orbital trick, and the multireference method NEVPT2(15e,11o),^[62]^ considering 15 active electrons and 11 active orbitals. While methods like EE‐EOM‐CCSD(t)(a)* and NEVPT2 are accurate but computationally expensive, TD‐DFT offers a more cost‐effective approach for interpreting experimental photoelectron spectroscopy data, particularly for larger molecules. The NEVPT2 method calculated the relative energy between the ground and excited states of neutral Si_3_Cu_3_. The vertical detachment energy was then determined by adding this relative energy to VDE_1_ obtained at the CCSD(T) level. Due to the closed‐shell electron configuration of Si_3_Cu_3_^−^, each fully occupied molecular orbital corresponds to a possible one‐electron detachment channel, leading to a final doublet state.

As shown in Table S1, all three methods yielded consistent VDE values. We selected the EOM‐CCSD(t)(a)* approach as a benchmark for comparison with the experimental data (see Table 1). The calculated VDE_1_ for the ptSi isomer (2.47 eV) closely agrees with the experimental value of 2.57 eV, while the VDE_1_ for the nearest isomer (2.31 eV) is significantly lower. Furthermore, the computed VDEs for the ptSi isomer correspond well with experimental bands A, B, and C, whereas those for the nearest isomer do not match the experimental data (see Table S1). Thus, we conclude that the experimental photoelectron spectra exclusively support the presence of the ptSi isomer in Si_3_Cu_3_^−^, with no detectable contribution from other isomers.

In summary, this combined study of theoretical calculations and photoelectron spectroscopy has identified the global minimum structure of Si_3_Cu_3_^−^. This 16‐valence electron cluster has a rhombic structure composed of triangular Si_3_ and Cu_3_ units interconnected through their Si_2_ and Cu_2_ edges in an ordered chain‐like arrangement, featuring a planar tetracoordinate silicon atom. In addition to the 2c–2e σ‐bond connecting the Si_3_ and Cu_3_ units, the electronic stability of this anion is significantly enhanced by a delocalized 3c–2e σ‐bond within Cu_3_ and a π‐bond within Si_3_. This cluster represents a rare example of an experimentally validated planar hypercoordinate atom system among the heavier Group 14 elements, opening exciting possibilities for extending these unique structures into two‐dimensional materials.

## Conflict of Interests

The authors declare no conflict of interest.

## Supporting information

As a service to our authors and readers, this journal provides supporting information supplied by the authors. Such materials are peer reviewed and may be re‐organized for online delivery, but are not copy‐edited or typeset. Technical support issues arising from supporting information (other than missing files) should be addressed to the authors.

Supporting Information

## Data Availability

The data that support the findings of this study are available in the supplementary material of this article.

## References

[1] R. Keese , Chem. Rev. 2006, 106, 4787–4808. 17165674 10.1021/cr050545h

[2] G. Merino , M. A. Méndez-Rojas , A. Vela , T. Heine , J. Comput. Chem. 2007, 28, 362–372. 17143864 10.1002/jcc.20515

[3] L. M. Yang , E. Ganz , Z. Chen , Z. X. Wang , P. v. R. Schleyer , Angew. Chem. Int. Ed. 2015, 54, 9468–9501. 10.1002/anie.201410407 26119555

[4] J. A. Le Bel , Bull. Soc. Chim. Fr. 1874, 22, 337–347.

[5] R. Hoffmann , R. W. Alder , C. F. Wilcox , J. Am. Chem. Soc. 1970, 92, 4992–4993.

[6] H. J. Monkhorst , Chem. Commun. (London) 1968, 1111.

[7] M. J. M. Pepper , I. Shavitt , P. v. R. Schleyer , M. N. Glukhovtsev , R. Janoschek , M. Quack , J. Comput. Chem. 1995, 16, 207–225.

[8] P. v. R. Schleyer , A. I. Boldyrev , J. Chem. Soc. Chem. Commun. 1991, 1536–1538.

[9] A. I. Boldyrev , J. Simons , J. Am. Chem. Soc. 1998, 120, 7967–7972.

[10] F. A. Cotton , M. Millar , J. Am. Chem. Soc. 1977, 99, 7886–7891.

[11] M. Albrecht , G. Erker , C. Krüger , Synlett 1993, 1993, 441–448.

[12] A. I. Boldyrev , J. Simons , X. Li , L.-S. Wang , J. Chem. Phys. 1999, 111, 4993–4998.

[13] X. Li , H.-F. Zhang , L.-S. Wang , G. D. Geske , A. I. Boldyrev , Angew. Chem. Int. Ed. 2000, 39, 3630–3632. 11091420

[14] S. D. Li , G. M. Ren , C. Q. Miao , Z. H. Jin , Angew. Chem. Int. Ed. 2004, 43, 1371–1373. 10.1002/anie.200353068 15368409

[15] Z.-h. Cui , M. Contreras , Y.-h. Ding , G. Merino , J. Am. Chem. Soc. 2011, 133, 13228–13231. 21793586 10.1021/ja203682a

[16] X. Li , L.-S. Wang , A. I. Boldyrev , J. Simons , J. Am. Chem. Soc. 1999, 121, 6033–6038.

[17] L.-M. Yang , Y.-H. Ding , C.-C. Sun , J. Am. Chem. Soc. 2007, 129, 658–665. 17227029 10.1021/ja066217w

[18] X. Li , H.-J. Zhai , L. S. Wang , Chem. Phys. Lett. 2002, 357, 415–419.

[19] C.-J. Zhang , W.-S. Dai , H.-G. Xu , X.-L. Xu , W. J. Zheng , J. Phys. Chem. A 2022, 126, 5621–5631. 35972885 10.1021/acs.jpca.2c04754

[20] C.-J. Zhang , P. Wang , X.-L. Xu , H.-G. Xu , W.-J. Zheng , Phys. Chem. Chem. Phys. 2021, 23, 1967–1975. 33470255 10.1039/d0cp06081j

[21] J. Xu , X. Zhang , S. Yu , Y.-H. Ding , K. H. Bowen , J. Phys. Chem. Lett. 2017, 8, 2263–2267. 28471673 10.1021/acs.jpclett.7b00732

[22] Y. Li , F. Li , Z. Zhou , Z. Chen , J. Am. Chem. Soc. 2011, 133, 900–908. 21182250 10.1021/ja107711m

[23] M.-J. Sun , X. Cao , Z. Cao , Nanoscale 2018, 10, 10450–10458. 29796564 10.1039/c8nr03566k

[24] P. Ghana , J. Rump , G. Schnakenburg , M. I. Arz , A. C. Filippou , J. Am. Chem. Soc. 2020, 143, 420–432. 33347313 10.1021/jacs.0c11628

[25] C. Shan , S. Dong , S. Yao , J. Zhu , M. Driess , J. Am. Chem. Soc. 2023, 145, 7084–7089. 36943751 10.1021/jacs.3c00722

[26] Y.-X. Li , L.-X. Bai , J.-C. Guo , Molecules 2023, 28, 5583.37513457

[27] B. Ding , R. Keese , H. Stoeckli-Evans , Angew. Chem. Int. Ed. 1999, 38, 375–376. 10.1002/(SICI)1521-3773(19990201)38:3<375::AID-ANIE375>3.0.CO;2-D 29711656

[28] A. I. Boldyrev , P. v. R. Schleyer , R. Keese , Mendeleev Commun. 1992, 2, 93–95.

[29] E. U. Würthwein , P. v. R. Schleyer , Angew. Chem. Int. Ed. Engl. 1979, 18, 553–554.

[30] W. Hönle , U. Dettlaff-Weglikowska , L. Walz , H. G. von Schnering , Angew. Chem. Int. Ed. Engl. 1989, 28, 623–624.

[31] D. Hartmann , T. Thorwart , R. Müller , J. Thusek , J. Schwabedissen , A. Mix , J.-H. Lamm , B. Neumann , N. W. Mitzel , L. Greb , J. Am. Chem. Soc. 2021, 143, 18784–18793. 34699725 10.1021/jacs.1c09746

[32] A. I. Boldyrev , X. Li , L.-S. Wang , Angew. Chem. Int. Ed. 2000, 39, 3307–3310. 10.1002/1521-3773(20000915)39:18<3307::aid-anie3307>3.0.co;2-# 11028086

[33] R. Islas , T. Heine , K. Ito , P. v. R. Schleyer , G. Merino , J. Am. Chem. Soc. 2007, 129, 14767–14774. 17983228 10.1021/ja074956m

[34] S.-D. Li , C.-Q. Miao , J. Phys. Chem. A 2005, 109, 7594–7597. 16834129 10.1021/jp0530000

[35] J.-C. Guo , S.-D. Li , J. Mol. Struct. 2007, 816, 59–65.

[36] S.-D. Li , G.-M. Ren , C.-Q. Miao , Inorg. Chem. 2004, 43, 6331–6333. 15446880 10.1021/ic049623u

[37] S.-D. Li , J.-C. Guo , C.-Q. Miao , G.-M. Ren , J. Phys. Chem. A 2005, 109, 4133–4136. 16833737 10.1021/jp050378p

[38] S.-D. Li , C.-Q. Miao , J.-C. Guo , G.-M. Ren , J. Am. Chem. Soc. 2004, 126, 16227–16231. 15584759 10.1021/ja045303y

[39] M.-H. Wang , X. Dong , Z.-h. Cui , M. Orozco-Ic , Y.-H. Ding , J. Barroso , G. Merino , Chem. Commun. 2020, 56, 13772–13775. 10.1039/d0cc06107g 33089264

[40] C. Chen , M.-H. Wang , L.-Y. Feng , L.-Q. Zhao , J.-C. Guo , H.-J. Zhai , Z.-h. Cui , S. Pan , G. Merino , Chem. Sci. 2022, 13, 8045–8051. 35919428 10.1039/d2sc01761j PMC9278486

[41] J.-C. Guo , C.-Q. Miao , G.-M. Ren , Comput. Theor. Chem. 2014, 1032, 7–11.

[42] J.-C. Guo , H.-X. Wu , G.-M. Ren , C.-Q. Miao , Y.-X. Li , Comput. Theor. Chem. 2016, 1083, 1–6.

[43] A. N. Alexandrova , M. J. Nayhouse , M. T. Huynh , J. L. Kuo , A. V. Melkonian , G. Chavez , N. M. Hernando , M. D. Kowal , C.-P. Liu , Phys. Chem. Chem. Phys. 2012, 14, 14815–14821. 22868353 10.1039/c2cp41821e PMC3478443

[44] L.-Q. Zhao , J.-C. Guo , H.-J. Zhai , Phys. Chem. Chem. Phys. 2022, 24, 7068–7076. 35258052 10.1039/d1cp05226h

[45] J. Xu , Y.-h. Ding , J. Comput. Chem. 2015, 36, 355–360. 25430676 10.1002/jcc.23792

[46] J.-J. Sui , J. Xu , Y.-H. Ding , Dalton Trans. 2016, 45, 56–60. 26605837 10.1039/c5dt03989d

[47] F. Ebner , L. Greb , J. Am. Chem. Soc. 2018, 140, 17409–17412. 30500194 10.1021/jacs.8b11137

[48] P. Ghana , J. Rump , G. Schnakenburg , M. I. Arz , A. C. Filippou , J. Am. Chem. Soc. 2021, 143, 420–432. 33347313 10.1021/jacs.0c11628

[49] C. Shan , S. Dong , S. Yao , J. Zhu , M. Driess , J. Am. Chem. Soc. 2023, 145, 7084–7089. 36943751 10.1021/jacs.3c00722

[50] F. Ebner , L. Greb , Chem 2021, 7, 2151–2159. 34435162 10.1016/j.chempr.2021.05.002 PMC8367297

[51] X.-b. Liu , Z.-h. Cui , Genetic Algorithm and Structure-driven Approaches for atomic clusters (GASA), Jilin University, 2024.

[52] C. Adamo , V. Barone , J. Chem. Phys. 1999, 110, 6158–6170.

[53] S. Grimme , J. Antony , S. Ehrlich , H. Krieg , J. Chem. Phys. 2010, 132, 154104. 20423165 10.1063/1.3382344

[54] F. Weigend , R. Ahlrichs , Phys. Chem. Chem. Phys. 2005, 7, 3297–3305. 16240044 10.1039/b508541a

[55] G. D. Purvis III , R. J. Bartlett , J. Chem. Phys. 1982, 76, 1910–1918.

[56] P. Pyykkö , M. Atsumi , Chem. Eur. J. 2009, 15, 186–197. 19058281 10.1002/chem.200800987

[57] D. Y. Zubarev , A. I. Boldyrev , Phys. Chem. Chem. Phys. 2008, 10, 5207–5217. 18728862 10.1039/b804083d

[58] T. Lu , F. Chen , J. Comput. Chem. 2012, 33, 580–592. 22162017 10.1002/jcc.22885

[59] A. Savin , R. Nesper , S. Wengert , T. F. Fässler , Angew. Chem. Int. Ed. Engl. 1997, 36, 1808–1832.

[60] M. E. Casida , D. R. Salahub , J. Chem. Phys. 2000, 113, 8918–8935.

[61] J. F. Matthews Devin , A. Stanton , J. Chem. Phys. 2016, 145, 124102. 27782677 10.1063/1.4962910

[62] C. Angeli , R. Cimiraglia , S. Evangelisti , T. Leininger , J. P. Malrieu , J. Chem. Phys. 2001, 114, 10252. 10.1063/1.2911699 18465905

