# Supplementary material for: Planar Tetracoordinate Silicon in Si3Cu3 − Cluster
Source: Angew Chem Int Ed Engl. 2024 Nov 7;64(3):e202415789. doi: 10.1002/anie.202415789 (PMC11735888; doi:10.1002/anie.202415789)
Supplement: Supplementary file 1 — Supporting Information [file ANIE-64-e202415789-s001.pdf]

Angewandte Chemie
Eine Zeitschrift der Gesellschaft Deutscher Chemiker

Supporting Information

Planar Tetracoordinate Silicon in $Si_3Cu_3^-$ Cluster

X.-h. Yin, H.-l. Zeng, X.-b. Liu, X.-L. Xu, H.-G. Xu, G. Merino, W.-j. Zheng*, Z.-h. Cui**

Supporting Information For
Planar Tetracoordinate Silicon in $Si_3Cu_3^-$ Cluster

Xiao-han Yin,[a#] Hong-lin Zeng,[b#] Xin-bo Liu,[a] Xi-Ling Xu,[b] Hong-Guang Xu,[b] Gabriel Merino,*[c] Wei-jun Zheng,*[b] Zhong-hua Cui*[a,d]

[a]*Institute of Atomic and Molecular Physics, Jilin University, Changchun 130023, China*

E-mail: zcui@jlu.edu.cn

[#b]*Beijing National Laboratory for Molecular Sciences, State Key Laboratory of Molecular Reaction Dynamics, Institute of Chemistry, Chinese Academy of Sciences, Beijing, China*

E-mail: zhengwj@iccas.ac.cn

[c]*Departamento de Física Aplicada, Centro de Investigación y de Estudios Avanzados, Unidad Mérida. Km 6 Antigua Carretera a Progreso. Apdo. Postal 73, Cordemex, 97310, Mérida, Yuc., México*

Email: gmerino@cinvestav.mx

[d]*Key Laboratory of Physics and Technology for Advanced Batteries (Ministry of Education), Jilin University, Changchun 130023, China*

[#]These authors contributed equally to this work.

I. Computational Methods

The structure searches were performed using the Genetic Algorithm and Structure-driven Approaches for atomic clusters program (GASA).[1] Initial structures for $Si_3Cu_3^-$ and $Si_3Cu_2^-$ in the first two spin states were optimized at the PBE0[2]-D3[3]/def2-SVP[4] level. For low-lying isomers (less than 20 kcal/mol above the global minimum), we employed the larger def2-TZVP[4] basis set. Single-point calculations for these isomers were performed at the CCSD(T,Full)[5]/def2-TZVP//PBE0/def2-TZVP level, with total energies corrected for the zero-point energies (ZPE). Natural bond orbital (NBO) calculations were carried out using NBO 7.0,[6-9] and Adaptive Natural Density Partitioning (AdDNP) analysis[10] was conducted with Multiwfn.[11] All these calculations were carried out with Gaussian 16.[12]

To compute higher detachment channels, we used several theoretical approaches, including time-dependent density functional theory (TD-DFT) methods[13] in Gaussian 16, and excited states equation-of-motion coupled-cluster single and double (EE-EOM-CCSD(t)(a)*)[14] methods through its unique treatment of the triples in CFOUR,[15] capturing both the ground and excited state correlation energies. Additionally, we employed the multireference method NEVPT2(15e,11o)[16-18] using 15 active electrons and 11 active orbitals, through ORCA[19] for comparison with experimental results.

II. Experimental Details

The experiments were conducted on a home-built apparatus described in detail elsewhere.[20] Briefly, the $Si_3Cu_3^-$ cluster was generated in a laser vaporization source by laser ablation of a rotating and translating disk target (13 mm diameter, Cu:Si mole ratio of 5:1) with second harmonic (532 nm) light pulses from a Nd:YAG laser (Continuum Surelite II-10). Helium gas at a background pressure of ~0.4 MPa was expanded into the source through a pulsed valve (General Valve Series 9) to cool the formed clusters. The cluster anions passing through a skimmer were extracted and accelerated by pulsed voltages applied to the extraction plates. The $Si_3Cu_3^-$ cluster was size-selected using a mass gate and decelerated by a momentum decelerator before

being photodetached by another laser beam at either 355 or 266 nm wavelengths. The detached photoelectrons were energy-analyzed using a magnetic-bottle photoelectron spectrometer, with photoelectron spectra calibrated againt known spectra of Pb^-, Bi^- and I^-.[21-23] The resolution of the magnetic-bottle photoelectron spectrometer was approximately 40 meV for electrons with 1 eV kinetic energy.

References

[1] X.-B. Liu, Z.-H. Cui, *Genetic Algorithm and Structure-driven Approaches for atomic clusters (GASA)*, Jilin University, **2024**.

[2] A. Carlo, B. Vincenzo, *J. Chem. Phys.* **1999**, *110*, 6158-6170.

[3] S. Grimme, J. Antony, S. Ehrlich, H. Krieg, *J. Chem. Phys.* **2010**, *132*, 154104.

[4] F. Weigend, R. Ahlrichs, *Phys. Chem. Chem. Phys.* **2005**, *7*, 3297.

[5] G. D. Purvis, R. J. Bartlett, *J. Chem. Phys.* **1982**, *76*, 1910-1918.

[6] K. B. Wiberg, *Tetrahedron* **1968**, *24*, 1083-1096.

[7] J. P. Foster, F. Weinhold, *J. Am. Chem. Soc.* **1980**, *102*, 7211-7218.

[8] A. E. Reed, F. Weinhold, *J. Chem. Phys.* **1983**, *78*, 4066-4073.

[9] A. E. Reed, R. B. Weinstock, F. Weinhold, *J. Chem. Phys.* **1985**, *83*, 735-746.

[10] D. Y. Zubarev, A. I. Boldyrev, *Phys. Chem. Chem. Phys.* **2008**, *10*, 5207-5217.

[11] T. Lu, F. Chen, *J. Comput. Chem.* **2012**, *33*, 580-592.

[12] M. J. Frisch, G. W. Trucks, H. B. Schlegel, S. G. E., M. A. Robb, J. R. Cheeseman, G. Scalmani, V. Barone, G. A. Petersson, H. Nakatsuji, X. Li, M. Caricato, A. V. Marenich, J. Bloino, B. G. Janesko, R. Gomperts, B. Mennucci, H. P. Hratchian, J. V. Ortiz, A. F. Izmaylov, J. L. Sonnenberg, D. Williams-Young, F. Ding, F. Lipparini, F. Egidi, J. Goings, B. Peng, A. Petrone, T. Henderson, D. Ranasinghe, V. G. Zakrzewski, J. Gao, N. Rega, G. Zheng, W. Liang, M. Hada, M. Ehara, K. Toyota, R. Fukuda, J. Hasegawa, M. Ishida, T. Nakajima, Y. Honda, O. Kitao, H. Nakai, T. Vreven, K. Throssell, J. A. Montgomery, Jr., J. E. Peralta, F. Ogliaro, M. J. Bearpark, J. J. Heyd, E. N. Brothers, K. N. Kudin, V. N. Staroverov, T. A. Keith, R. Kobayashi, J. Normand, K. Raghavachari, A. P. Rendell, J. C. Burant, S. S. Iyengar, J. Tomasi, M. Cossi, J. M. Millam, M. Klene, C. Adamo, R. Cammi, J. W. Ochterski, R. L. Martin, K. Morokuma, O. Farkas, J. B. Foresman, D. J. Fox, *Gaussian 16, Revision C.01*, Gaussian. Inc., Wallingford, CT, **2019**.

[13] M. E. Casida, D. R. Salahub, *J. Chem. Phys.* **2000**, *113*, 8918-8935.

[14] J. F. Matthews Devin A.; Stanton, *J. Chem. Phys.* **2016**, *145*, 124102.

[15] D. A. C. Matthews, Lan; Harding, Michael E.; Lipparini, Filippo; Stopkowicz, Stella; Jagau, Thomas- C.; Szalay, Péter G.; Gauss, Jürgen; Stanton, John F., *J. Chem. Phys.* **2020**, *152*, 214108.

[16] C. C. Angeli, R.; Malrieu, J. P., *J. Chem. Phys.* **2002**, *117*, 9138-9153.

[17] C. C. Angeli, Renzo; Malrieu, Jean-Paul, *Chem. Phys. Lett.* **2001**, *350*, 297-305.

[18] C. Angeli, R. Cimiraglia, S. Evangelisti, T. Leininger, J. P. Malrieu, *J. Chem. Phys.*

2001, *114*, 10252-10264.
[19] F. Neese, *Wires Comput Mol Sci* **2012**, *2*, 73-78.
[20] H.-G. Xu, Z.-G. Zhang, Y. Feng, J. Yuan, Y. Zhao, W. Zheng, *Chem. Phys. Lett.* **2010**, *487*, 204-208.
[21] X. Chen, C. Ning, *J. Chem. Phys.* **2016**, *145*, 084303.
[22] J. C. Rienstra-Kiracofe, G. S. Tschumper, H. F. Schaefer, S. Nandi, G. B. Ellison, *Chem. Rev.* **2002**, *102*, 231-282.
[23] J. E. Sansonetti, W. C. Martin, *J. Phys. Chem. Ref. Data* **2005**, *34*, 1559-2259.

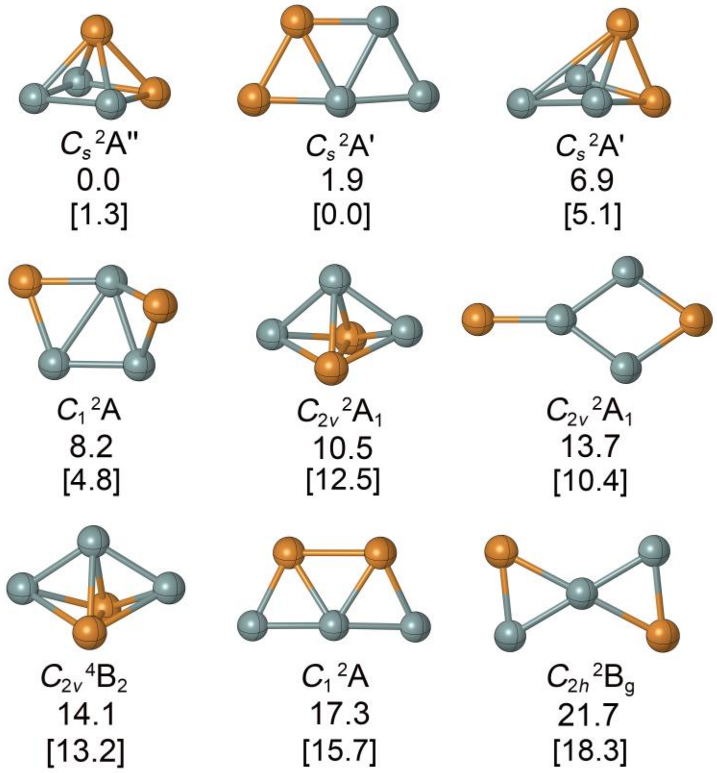

Figure S1. Structures and relative energies (in kcal/mol) of the low-lying isomers of $Si_3Cu_2^-$, calculated at the CCSD(T)/def2-TZVP//PBE0-D3/def2-TZVP level. The total energy values are corrected for zero-point energy at the PBE0-D3/def2-TZVP level. Relative energies in square brackets correspond to those calculated using PBE0-D3/def2-TZVP. Orange and gray spheres represent Cu and Si atoms, respectively.

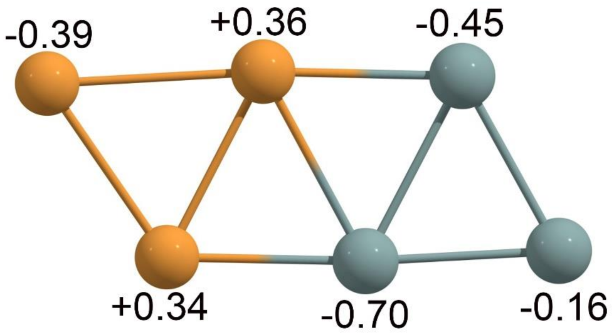

Figure S2. NPA charges (in |e|) of the ptSi $Si_3Cu_3^-$ clusters, computed at the PBE0-D3/def2-TZVP level. Orange and gray spheres represent Cu and Si atoms, respectively.

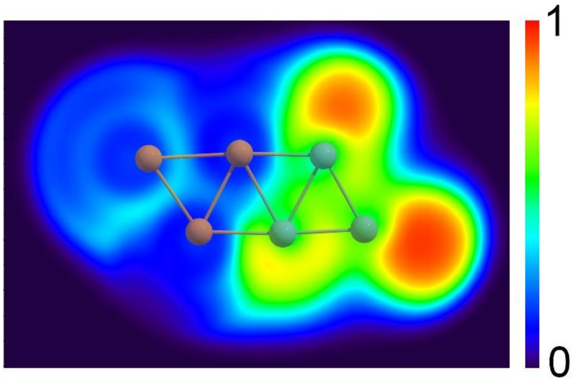

Figure S3. Electron localization function (ELF) of $Si_3Cu_3^-$ calculated at a height of 1 Å above the molecular plane using the PBE0-D3/def2-TZVP level.

Table S1. Theoretical VDEs of two low-lying $Si_3Cu_3^-$ isomers calculated using the TD-PBE0-D3, EE-EOM-CCSD(t)(a)*, and NEVPT2(15e,11o) methods with the def2-TZVP basis set, compared to experimental VDEs. The higher VDEs from the NEVPT2(15e,11o) method are based on VDE_1 values calculated using the CCSD(T) method.

Peak	VDE (eV) (Expt.)	Final state	Electronic configuration	VDE (eV) (Theor.)		
				PBE0	EOM-CCSD(t) (a)*	NEVPT2(15,11)
ptSi						
X	2.57	$^2A'$	13a'^{2}7a"^{2}14a'^{2}15a'^{2}**16a'**^{1}	2.51	2.47	2.55
A	2.74	$^2A'$	13a'^{2}7a"^{2}14a'^{2}**15a'**^{1}16a'^{2}	3.17	2.98	3.09
B	3.32	$^2A'$	13a'^{2}7a"^{2}**14a'**^{1}15a'^{2}16a'^{2}	3.44	3.36	3.51
C	3.58	$^2A''$	13a'^{2}**7a"**^{1}14a'^{2}15a'^{2}16a'^{2}	3.60	3.52	3.76
the second lowest-energy 3D isomer						
X	2.57	$^2A''$	12a'^{2}8a"^{2}13a'^{2}14a'^{2}**9a"**^{1}	2.33	2.31	2.37
A	2.74	$^2A'$	12a'^{2}8a"^{2}13a'^{2}**14a'**^{1}9a"^{2}	2.72	2.62	2.68
B	3.32	$^2A'$	12a'^{2}8a"^{2}**13a'**^{1}14a'^{2}9a"^{2}	3.24	2.91	2.96
C	3.58	$^2A''$	12a'^{2}**8a"**^{1}13a'^{2}14a'^{2}9a"^{2}	3.99	3.81	4.09

III. Cartesian coordinates of $Si_3Cu_3^-$ at PBE0-D3/def2-TZVP level

$Si_3Cu_3^-$

Si	3.527145000	-0.899907000	0.000000000
Si	1.311614000	-1.137127000	0.000000000
Si	2.291669000	1.051829000	0.000000000
Cu	-0.972705000	-1.213496000	0.000000000
Cu	-2.469570000	0.709203000	0.000000000
Cu	0.000000000	0.979909000	0.000000000
